# Distribution of perivascular spaces distribution and relate to the clinical features of SCA3

**DOI:** 10.1186/s13023-025-03954-3

**Published:** 2025-08-11

**Authors:** Xinyuan Chen, Yanhua Lian, Wei Lin, Xiaoyue Xia, Lin Zhang, Zhuoying Huang, Maolin Cui, Ruying Yuan, Mengcheng Li, Ziqiang Huang, Naping Chen, Yuqing Tu, Jianping Hu, Ning Wang, Qunlin Chen, Shirui Gan

**Affiliations:** 1 Department of Rehabilitation Medicine, The First Affiliated Hospital, Fujian Medical University, Fuzhou, 350005 China; 2 Fuzhou Second General Hospital, Fuzhou, 350007 China; 3 Department of Neurology, Institute of Neurology of First Affiliated Hospital, Institute of Neuroscience, Fujian Key Laboratory of Molecular Neurology, Fujian Medical University, Fuzhou, 350005 China; 4 Department of Radiology, The First Affiliated Hospital, Fujian Medical University, Fuzhou, 350005 China; 5 National Regional Medical Center, Binhai Campus of the First Affiliated Hospital, Fujian Medical University, Fuzhou, 350212 China

## Abstract

**Background:**

Spinocerebellar ataxia type 3 (SCA3) is a rare neurodegenerative condition. Prior research has established perivascular spaces (PVS) expansion has been implicated in the pathogenesis and prognosis of various neurodegenerative diseases. To examine PVS changes in SCA3 by comparing patients and matched healthy controls and to identify potential connection of PVS for clinical features.

**Methods:**

We conducted MRI scans on 91 SCA3 patients and 64 healthy controls. We utilized visual semi-quantitative methods to assess PVS in various brain regions, including the center of the semiovale (CSO), basal ganglia (BG), and midbrain-pons, as well as combinations (BG + CSO, BG + CSO + midbrain-pons). To differentiate SCA3 patients from healthy controls, we compared the area under the curve (AUC) of the receiver operating characteristic curve between the two groups. Additionally, we employed Pearson’s correlation coefficient to examine the relationship between PVS scores in each brain region and clinical indicators among SCA3 patients.

**Results:**

In the SCA3 group, we observed higher levels of PVS in the BG, CSO + BG, and three brain regions compared to the healthy control group. PVS in the CSO and CSO + BG also showed positive correlations with age and disease duration, and negative correlations with the number of CAG repeats. Furthermore, PVS in three brain regions exhibited negative correlation with the number of CAG repeats.

**Conclusions:**

This study represents an initial investigation into the relationship between PVS and SCA3 disease. Our findings suggest that PVS might indicate the degree of cerebellar ataxia to a certain extent.

## Introduction

Spinocerebellar ataxia type 3 (SCA3) stands as a rare inherited neurodegenerative condition, representing the predominant autosomal dominant spinocerebellar ataxia [1]. It emerges from a CAG amplification mutation within the ATXN3 gene, prompting a structural alteration in the associated protein and leading to its dysfunctional state. The aggregation of aberrant proteins within neurons can instigate neurotoxic effects, fostering neuronal degeneration, demyelination, and consequent structural brain atrophy [2]. SCA3 manifests with characteristic features such as postural instability, irregularities in ocular muscle movements, dysarthria, and peripheral neuropathy [3]. Previous investigations via neuroimaging and pathological analyses have confirmed widespread neurodegeneration across various brain regions, including the cerebellum, brainstem, basal ganglia, thalamus, and cerebral cortex, in individuals afflicted with SCA3 [4].

The perivascular space (PVS), also termed the Virchow-Robin space, encompasses the fluid-filled area encircling blood vessels as they traverse from the subarachnoid space through the brain parenchyma [5]. Predominantly observed in the center of the semiovoid (CSO) and basal ganglia (BG) [6], PVS plays a pivotal role in eliminating interstitial waste products from the brain [7] and modulating immune responses. Although typically small, dilated PVS can be discerned using routine magnetic resonance imaging (MRI) sequences. The mechanism underlying PVS dilation remains unclear, with some scholars proposing associations with cerebral atrophy, disruption of the blood-brain barrier, impaired interstitial fluid drainage, and inflammation [8–10]. Notably, heightened PVS levels have been implicated in protein misfolding disorders like Alzheimer’s disease (AD) [11, 12], Huntington’s disease (HD) [13, 14], and Parkinson’s disease (PD) [15, 16]. PVS expansion may contribute to neurodegeneration either by insufficient clearance of neurotoxic proteins or by occupying the space created by brain atrophy [17–19]. Given that SCA3 is pathogenic due to protein misfolding, similar mechanisms are shared with AD and PD. We believe that PVS expansion may also manifest in SCA3. However, the precise distribution of PVS in SCA3 remains elusive, and the association between PVS and diminished ataxia function has yet to be explored.

Our main objective was to compare the presence of PVS in different brain regions between SCA3 patients and healthy controls. We aimed to assess if the severity of PVS in five regions (BG, CSO, midbrain-pons, BG + CSO, BG + CSO + midbrain-pons) correlates with decreased ataxia function in SCA3. Additionally, we sought to investigate if the extent of PVS severity in these regions correlates with patient age, disease duration, and the number of CAG repetitions.

## Method

### Participants

Standard Protocol Approvals, Registrations, and Patient Consents.

Approval for this study was granted by the Ethics Committee for Medical Research of the First Affiliated Hospital of Fujian Medical University (approval numbers: [2019]120 and [2020]431). The study was carried out in accordance with the Declaration of Helsinki (1964). Written informed consent was obtained from all participants. Patients diagnosed with SCA3 were recruited from the Organization in South-East China for Cerebellar Ataxia Research (OSCCAR), specifically those diagnosed at the First Affiliated Hospital of Fujian Medical University between February 2019 and October 2022. The study was retrospectively conducted. This study is registered with ClinicalTrials.gov under ID number NCT04010214.

### SCA3 group

Inclusion criteria included: (1) SCA3 patients confirmed through genetic testing with a SARA score ≥ 3; (2) provision of informed consent by patients, documented by signed informed consent form.

Exclusion criteria included: (1) presence of major central nervous system (CNS) or psychiatric disorders, such as stroke, multiple sclerosis (MS), AD, PD, HD, CNS inflammation, developmental disorders, schizophrenia, bipolar disorder, motor neuron disease, or any other conditions that might affect assessment; (2) contraindications to MRI examination (e.g., individuals with cardiac pacemakers, those in early pregnancy beyond the third trimester, and individuals experiencing claustrophobia, etc.); and (3) inadequate quality of magnetic resonance images.

### Healthy control group

Inclusion criteria included: (1) absence of notable central nervous system disease or mental illness; (2) patients consenting and signing informed consent forms.

Exclusion criteria included: (1) presence of central nervous system diseases, psychiatric disorders, or other conditions potentially influencing assessment; (2) contraindications to MRI examination; (3) inadequate quality of magnetic resonance images.

Prior to enrollment, all participants underwent a thorough neurological examination administered by a skilled neurologist. Each subject provided a detailed medical history, including information such as age, sex, presence of hypertension, diabetes, hyperlipidemia, smoking habits, etc. Additionally, data regarding CAG repeat length/number of repetitions was collected [20]. Severity of cerebellar ataxia in SCA3 patients was evaluated using the Scale for the Assessment and Rating of Ataxia (SARA) scores [21], and the International Cooperative Ataxia Rating Scale (ICARS) scores [22]. Polymerase Chain Reaction (PCR) coupled with Sanger sequencing was employed to determine the CAG repeat number. Higher SARA and ICARS scores indicated more pronounced ataxia. Patients diagnosed with SCA3 in the symptomatic group exhibited clinically significant symptoms of cerebellar ataxia, defined as a SARA score ≥ 3.

Diagnostic criteria for PVS risk factors.

Research indicates that the enlargement of the PVS may correlate with various health conditions such as high blood pressure [5, 19], diabetes [5, 23], and smoking habits [24].

Hypertension is defined as having a systolic blood pressure of ≥ 140 mm Hg and/or diastolic blood pressure of ≥ 90 mm Hg on three separate measurements, not taken on the same day, without the use of antihypertensive medication, or having received a previous medical diagnosis of hypertension.

Diabetes mellitus is characterized by a random blood glucose level of ≥ 11.1 mmol/L, fasting blood glucose of ≥ 7.0 mmol/L, a glucose level of ≥ 11.1 mmol/L on a glucose tolerance test, or having received a previous medical diagnosis of diabetes.

Smoking status is determined through participant self-report, defined as smoking more than one cigarette per day for six consecutive or cumulative months.

### Image acquisition

All participants underwent neuroimaging using a Siemens 3.0T Skyra scanner equipped with a 20-channel head and neck coil (Germany, Siemens). To maintain image quality and minimize head movement, a sponge head fixation support was employed within the coil.

The neuroimaging protocol comprised the following sequences and parameters: (1) three-dimensional T1-weighted (3D-T1W) magnetization-prepared rapid gradient-echo (MP-RAGE) acquisition in the sagittal plane: Repetition time (TR) = 2300 ms, Echo time (TE) = 2.3 ms, Field of view (FOV) = 220 mm × 200 mm, Matrices = 240 × 256, Voxel size = 1 mm × 1 mm × 1 mm, Slice thickness = 1 mm; (2) T2-weighted fluid-attenuated inversion recovery (FLAIR) sequence: TR = 8500 ms, TE = 81 ms, FOV = 220 mm × 200 mm, Matrix size = 203 mm × 320 mm, Voxel size = 0.7 mm × 0.7 mm × 5 mm, Slice thickness = 5 mm.

According to the Standards for Reporting Vascular Changes in Neuroimaging (STRIVE) criteria, PVS are defined as fluid-filled cavities accompanying the brain’s penetrating vessels. Consequently, PVS exhibit cerebrospinal fluid signal intensity across magnetic resonance sequences, presenting as low signal in both 3D T1 and T2-FLAIR sequences. Typically, PVS manifest as round, oval, or linear cavities, typically measuring less than 3 mm in diameter.

### Analytical plan

During image analysis, distinguishing lacunar foci from PVS proved crucial, mainly considering their size and signal characteristics. Lacunar foci, typically ≥ 3 mm in diameter, exhibited a high signal ring discernible in T2-FLAIR sequences. We focused on three primary regions: the BG, CSO, and midbrain-pons.

In the BG and CSO, we conducted assessments at all relevant anatomical levels, separately evaluating each cerebral hemisphere. In cases of hemispheric asymmetry, we tallied PVS based on the hemisphere with a higher count. Subsequently, PVS in the CSO and BG were graded according to Potter [25], based on the quantity: 0 (no PVS), 1 (1–10 PVS), 2 (11–20 PVS), 3 (21–40 PVS), and 4 (> 40 PVS). For the midbrain-pons, PVS were categorized as either 0 (not visible) or 1 (visible). Ultimately, scores for PVS in the CSO, BG, and midbrain-pons were summed to yield a combined score for the three brain regions, ranging from 0 to 9. Image interpretation and analysis were conducted using Radiant software by two neuroimaging physicians blinded to the clinical data.

### Statistical analysis

We performed statistical analyses on all data utilizing IBM SPSS software (version 25.0). Normality tests were conducted using Shapiro-Wilk. Categorical variables were presented as counts (percentage), while continuous variables were expressed as means (standard deviation, SD). To compare baseline characteristics, we employed χ2 tests and Fisher’s exact tests for categorical variables. Additionally, analyses of covariance (ANCOVAs) were utilized to compare the two datasets, with smoking status serving as a covariate, use Welch’s test when the variance is not homogeneous. Correlations between PVS and various clinical indicators were evaluated using Spearman’s correlation coefficient. We assessed the accuracy of EPVS in determining disease severity through Receiver Operating Characteristic (ROC) curves and calculated the Area Under the Curve (AUC). For all statistical analyses mentioned above, a significance level of *p* < 0.05 was applied.

## Results

### Comparison of demographic and clinical data between groups

Table 1 presents the baseline clinical characteristics of both SCA3 patients and healthy controls. The SCA3 cohort consisted of 91 individuals, with an average age of 41.73 ± 10.10 years, among whom 54 (59.34%) were male. In contrast, the healthy control group comprised 64 individuals, with an average age of 41.09 ± 13.02 years, and 32 (50.00%) were male. Detailed baseline data are provided in Table 1.

**Table 1:** Demographic and clinical characteristics of SCA3 patients and healthy controls

	SCA3 group (*n* = 91)	Healthy control group (*n* = 64)	*p* value
Age (years)	41.73 ± 10.10	41.09 ± 13.02	0.735
Sex (male)	54 (59.34)	32 (50.00)	0.256
Hypertension n (%)	11 (12.09)	5 (7.81)	0.435
Diabetes mellitus n (%)	2 (2.20)	3 (4.69)	0.405
Hyperlipidaemia n (%)	0 (0.00)	1 (1.56)	0.413
Smoking n (%)	23 (25.27)	6 (9.38)	**0.013**
SARA score	9.92 ± 4.53	-	-
ICARS score	26.38 ± 12.63	-	-
Number of expanded CAG repeats	75.09 ± 3.09	-	-

Note: CAG = Cytosine-Adenine-Guanine; SARA = Scale of Ataxia Rating; ICARS = International Collaborative Ataxia Rating Scale

There were no statistically significant differences observed between the healthy control and SCA3 groups concerning age (*p* = 0.735), gender (*p* = 0.256), hypertension (*p* = 0.435), diabetes mellitus (*p* = 0.405), and hyperlipidemia (*p* = 0.413).

### Comparison of PVS scores between groups of different brain regions

**Fig. 1:**
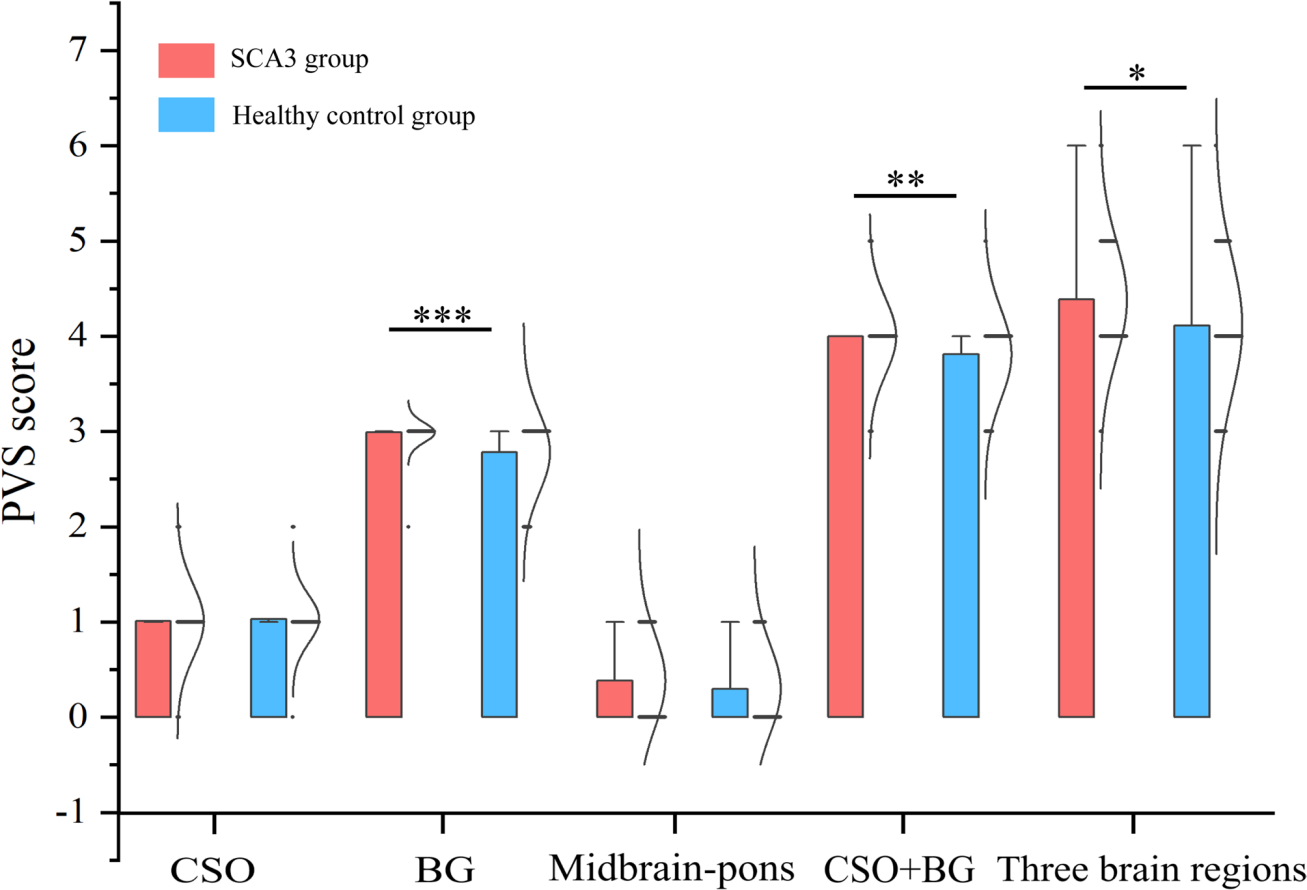
Between-group comparisons of PVS scores for each brain region. We analysed covariance (ANCOVA) to compare the two datasets, with smoking status as a covariate, and Welch’s test when the variance was uneven. Result showed statistically significant differences in PVS scores in the BG (*p* < 0.001, *p* = 0.000), as well as in the CSO + BG, for three brain regions (*p* < 0.05, *p* = 0.008, *p* = 0.022). Differences in the remaining regions were not statistically significant (*p* > 0.05). SCA3 = Spinocerebellar Ataxia Type 3; CSO = Centre of the Semiovoid; BG = Basal Ganglia. * *p* < 0.05, ** *p* < 0.01, *** *p* < 0.001

A significant discrepancy in smoking status (*p* = 0.013) was noted between the symptomatic and healthy control groups. We therefore analyzed covariance (ANCOVA) to compare the two datasets and used smoking status as a covariate, using Welch’s test when the variance was not homogeneous. After controlling for smoking confounders, we observed that PVS scores were significantly higher in the SCA3 group than in the healthy control group in the BG brain region (*p* < 0.001). In the CSO + BG brain region, the PVS score in the SCA3 group was significantly higher than that in the healthy control group (*p* = 0.008). The PVS score of the SCA3 group was significantly higher than that of the healthy control group in the three brain regions, and the difference was statistically significant (*p* = 0.022). Conversely, in the CSO and midbrain-pons regions, the disparity in PVS scores between groups did not reach statistical significance (*p* > 0.05). Detailed results are presented in Table 2; Fig. 1.

**Table 2:** PVS scores of different brain regions in the SCA3 group and in healthy controls

Brain region	SCA3 group	Healthy control group	*p* value
CSO	1.01 ± 0.38	1.03 ± 0.25	0.955
BG	2.99 ± 0.11	2.78 ± 0.42	**0.000**
Midbrain-pons	0.38 ± 0.49	0.30 ± 0.46	0.257
Two brain regions (BG + CSO)	4.0 ± 0.39	3.81 ± 0.47	**0.003**
Three brain regions (BG + CSO + midbrain-pons)	4.38 ± 0.61	4.11 ± 0.74	**0.005**

Abbreviations: CSO = Centre of the Semiovoid; BG = Basal Ganglia. SCA3 = Spinocerebellar Ataxia Type 3. Comparison between groups at midbrain-brain bridge sites using Welch’s test

### Subject ROC curves to determine the optimal cut-off value for disease

**Fig. 2:**
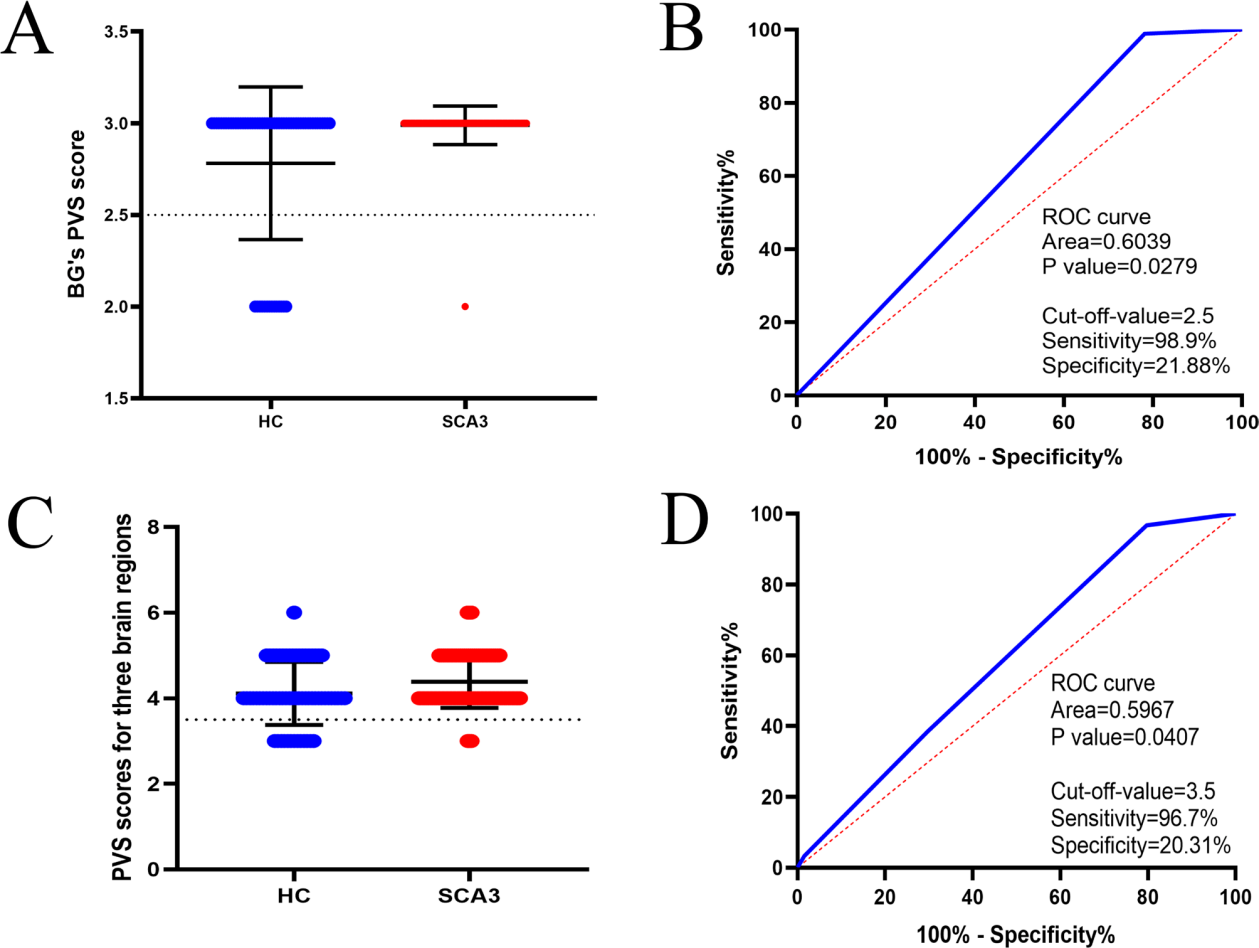
Area under the curve (AUC) of the operating characteristics in the BG for three brain regions for two groups. Variability of PVS scores in BG brain regions (AUC = 0.604; sensitivity = 0.989; specificity = 0.219, *p* = 0.028) and variability of PVS scores in three brain regions (AUC = 0.597; sensitivity = 0.967; specificity = 0.203, *p* = 0.041). SCA3 = Spinocerebellar Ataxia Type 3; HC = Healthy Control; BG = Basal Ganglia; ROC = Receiver Operating Characteristic

The ROC curve provides a comprehensive assessment of a diagnostic test’s performance, plotting the false-positive rate against the true-positive rate for various cut-off points of the test result. This curve aids in determining the optimal cut-off value for diagnosing a disease. The AUC value, representing the overall performance of the diagnostic test, is calculated as the average sensitivity across all possible specificities. Our analysis (Fig. 2) demonstrates that scores from the BG (AUC = 0.604; sensitivity = 0.989; specificity = 0.219, *p* = 0.028) and three other brain regions (AUC = 0.597; sensitivity = 0.967; specificity = 0.203, *p* = 0.041) offer superior predictive capability for SCA3 compared to scores from other brain regions. Particularly, the AUC value for the BG region was the highest, indicating its superior ability to predict the presence of SCA3 disease. The ROC curve serves as a valuable tool for visually discerning between normal and abnormal conditions across the entire spectrum of test results, aiding in disease diagnosis based on designated cut-off values. It plays a crucial role in predicting the likelihood of SCA3 disease in a patient.

### SCA3 group correlation analysis

We employed Pearson correlation analysis to investigate the relationship between CSO, BG, midbrain-pons, two brain regions, and three brain regions’ PVS scores with age, disease duration, number of CAG repetitions, SARA, and ICARS in the SCA3 group. Our statistical analysis unveiled several significant findings.

Firstly, a positive correlation emerged between CSO-PVS score and age (*r* = 0.409, *p* = 0.000) as well as disease duration (*r* = 0.243, *p* = 0.020), this indicates that as age increases, so does the CSO-PVS score, and similarly, with prolonged disease duration. Conversely, a negative correlation was observed with the number of CAG repetitions (*r* = -0.351, *p* = 0.001), suggesting a decline in the CSO-PVS score as the number of CAG repetitions increases. Secondly, the total score of two brain regions exhibited positive correlations with age (*r* = 0.421, *p* = 0.000) and disease duration (*r* = 0.236, *p* = 0.024), indicating that these scores increase with advancing age and prolonged disease duration. Conversely, a negative correlation was noted with the number of CAG repetitions (*r* = -0.356, *p* = 0.001), implying a decrease in the total score of two brain regions as the number of CAG repetitions rises. Furthermore, the total score of the three brain regions displayed a negative correlation with the number of CAG repetitions (*r* = -0.260, *p* = 0.013). In the healthy control group, CSO-PVS (*r* = 0.292, *p* = 0.019) and Midbrain-pons (*r* = 0.832, *p* = 0.027) scores were also positively correlated with age (*r* = 0.292, *p* = 0.019). However, no significant correlation was observed with the remaining indicators (Table 3).

**Table 3:** Analysis of the correlation between PVS scores and clinical indicators in various brain regions

Brain region	Disease duration	CAG	SARA	ICARS	SCA3 group age	HC group age
CSO	*r* = 0.243	*r* = -0.351	*r* = 0.110	*r* = 0.145	*r* = 0.409	*r* = 0.292
	***p =*** **0.020**	***p =*** **0.001**	*p* = 0.297	*p* = 0.170	***p =*** **0.000**	***p*** **= 0.019**
BG	*r* = 0.006	*r* = -0.066	*r* = 0.021	*r* = 0.045	*r* = 0.102	*r* = -0.178
	*p* = 0.953	*p* = 0.536	*P* = 0.840	*p* = 0.670	*p* = 0.336	*p* = 0.160
Midbrain-pons	*r* = -0.064	*r* = -0.037	*r* = 0.045	*r* = 0.003	*r* = -0.089	*r* = 0.832
	*p* = 0.547	*p* = 0.725	*p* = 0.675	*p* = 0.979	*p* = 0.404	***p*** **= 0.027**
Two brain regions (BG + CSO)	*r* = 0.236	*r* = -0.356	*r* = 0.112	*r* = 0.152	*r* = 0.421	*r* =-0.002
	***p =*** **0.024**	***p*** **= 0.001**	*p* = 0.290	*p* = 0.151	***p =*** **0.000**	*p* = 0.986
Three brain regions (BG + CSO + midbrain-pons)	*r* = 0.101	*r* = -0.260	*r* = 0.108	*r* = 0.100	*r* = 0.201	*r* = 0.015
	*p* = 0.340	***p*** **= 0.013**	*p* = 0.308	*p* = 0.344	***p =*** **0.056**	*p* = 0.904

Abbreviations: CSO = Centre of the Semiovoid; BG = Basal Ganglia; SARA = Scale for the Assessment and Rating of Ataxia; ICARS = International Cooperative Ataxia Rating Scale; CAG = Cytosine-Adenine-Guanine; HC group = Healthy control group

## Discussion

In recent years, there has been growing interest in studying the structural and magnetic resonance imaging of the brain associated with PVS. However, the distribution of PVS in SCA3 remains poorly understood, and the correlation between PVS and SCA3 has not been explored. Thus, we undertook a preliminary investigation to explore this relationship. Our study uncovered several significant findings. Our analysis suggests that PVS may serve as a partial indicator of the pathological characteristics of SCA3. Specifically, we found that the BG-PVS score could aid in distinguishing between individuals with SCA3 and those who are healthy.

The PVS primarily functions to eliminate interstitial fluids and cerebral wastes [17]. This process is intricately linked with the perineural lymphatic drainage, blood-brain barrier (BBB) [17, 18], and neurodegenerative changes [26]. The PVS expansion indicates that the cellular debris and other wastes are produced around blood vessels, and they then lead to the dysfunctions of cerebrovascular reactivity (CVR) and BBB, thus finally reducing the waste proteins’ clearance from interstitial fluid (ISF). This cascade ultimately leads to the accumulation of tissue damage, toxins, and hypoxia [10].

Notably, PVS expansion has been implicated in the pathogenesis and prognosis of various neurodegenerative diseases including AD [27], MS [7, 28, 29], PD [16, 30], and HD [13]. For instance, in patients with HD, PVS expansion may be influenced by brain atrophy and is associated with early cognitive decline [31]. Similarly, in Parkinson’s disease patients, an enlarged PVS may indicate poor drainage of α-synuclein, contributing to gait disturbances [32].

SCA3, primarily affecting the cerebellum, presents varied pathological changes across different brain regions. Our investigation highlights the association between PVS loading and the pathogenesis of SCA3, particularly concentrated in the BG areas. We propose that BG-PVS is closely linked to ATXN3 protein aggregation, vascular barrier disruption, and neuroinflammation. The heightened burden of PVS may also indicate neuroinflammation, ultimately culminating in neuronal loss [7]. Previous research indicates that in SCA3, neuronal loss predominantly occurs in the dentate nucleus and basal ganglia [33] rather than the cerebellar cortex. The progression of SCA3 is intertwined with demyelination, inflammatory changes [34], and evident atrophy in multiple brain regions, including the basal ganglia, brainstem, and cerebellum. The aggregation of ATXN3 protein within the cerebral vasculature in SCA3 patients, coupled with disruptions in the blood-brain barrier, likely contributes to decreased PVS clearance, leading to its enlargement.

Experimental findings by Yue Li [8] and colleagues support the notion that the enlargement of BG-PVS is associated with compromised blood-brain barrier integrity. This aligns with the hypothesis that blood-brain barrier dysfunction plays a role in the pathogenesis of BG-PVS enlargement. Disruption of the blood-brain barrier is a common feature across various neurodegenerative diseases [34]. Furthermore, studies employing immunofluorescence techniques and magnetic resonance dynamic contrast enhancement assessment in SCA3 transgenic mouse models have demonstrated blood-brain barrier disruption, a finding corroborated in postmortem brain samples from SCA3 patients [34].

Our Pearson correlation analysis in this study uncovered a mild positive relationship between CSO-PVS and age in SCA3 patients, along with a similar positive correlation between PVS in diencephalic regions and age. Past investigations have consistently highlighted age [17, 35, 36] and gender [17] as reliable predictors of PVS expansion [37]. There is a notable trend of PVS enlargement with advancing age, particularly evident in the basal ganglia region [38]. Previous visual scoring studies have also noted correlations between age and PVS in the basal ganglia [26, 39], echoing our findings. Additionally, our results found a correlation between CSO and midbrain PVS scores and age in healthy controls. Compared to the normal population, PVS scores in SCA3 patients had a stronger correlation with age, but there was no significant difference between the two, which may be related to the fact that SCA3 disease does not cause a pathological increase in PVS in the CSO. Zong X observed a tendency of increased apparent diameter of the PVS with age in the basal ganglia and midbrain of healthy adults [40]. A recent meta-analysis [41] further solidified the strong association between PVS and age. Taken together, these studies suggest that PVS expansion may reflect processes associated with brain aging [5, 35]. Factors such as perivascular inflammation and age-related changes likely contribute to the accumulation of metabolic wastes within the PVS, resulting in its dilation and thickening [6, 10, 42].

Increased PVS load in SCA patients may be the result of a combination of pathological mechanisms, including neuroinflammation [34], smoking and sleep disturbances [43]. Neuroinflammatory responses are common in neurodegenerative diseases, and SCA3 is no exception. When inflammation occurs in the CNS, vascular permeability increases, increasing the amount of substances entering the PVS and increasing its clearance load, which reduces its ability to remove metabolites and ultimately leads to enlargement of the PVS. In addition, inflammation may in turn affect the blood-brain barrier, exacerbating its disruption. Compared to normal subjects, SCA3 patients have a higher prevalence of smoking, and smoking also affects the PVS score, which in turn affects the correlation of PVS with SARA and ICARS. In this study, PVS scores of SCA3 patients in CSO, CSO + BG and three brain regions were negatively correlated with CAG repeat length/number. Previous studies have shown that CAG expansion reflects disease severity in SCA3 patients, i.e. the higher the number of CAG repeats, the more severe the disease, but CAG repeat length/number does not fully explain all variations in disease severity. Other factors such as genetic polymorphisms, environment [44], lifestyle [45], fatigue [46], body mass index BMI [47], etc., may contribute to the irrelevance of PVS scores to disease severity. Therefore, in the present small sample study, it is possible that a variety of these factors may have contributed to the lack of a significant correlation between PVS and SCA3 disease severity. Previous studies have more frequently confirmed that there is a strong association between PVS and the cognitive impairment of the disease, but the relationship between PVS and motor dysfunction, particularly ataxia, is not clear. Therefore, there is a need to explore the intrinsic links using larger SCA3 population cohorts as well as animal models.

This study marks the inaugural exploration into the relationship between PVS and SCA3 disease. Our findings suggest that PVS may serve as an indicator of cerebellar ataxia severity to some degree. Notably, the BG-PVS score and the PVS scores across three brain regions show promise in predicting SCA3 disease. However, several limitations warrant acknowledgment. Firstly, the predominantly retrospective nature of our study resulted in the inclusion of only 3D-T1W and T2-FLAIR sequences, overlooking the potentially more sensitive T2WI sequences for fluid signal detection. Future investigations will incorporate T2WI sequences to bolster imaging evidence. Secondly, SCA3 disease being a rare inherited degenerative condition, our sample size remains relatively small, potentially compromising the study’s representativeness. To mitigate this limitation, we propose future endeavors to engage in multi-center collaborative studies, thus expanding the sample size for more robust conclusions.

## Conclusion

This study represents the inaugural examination of PVS load in SCA3 patients. Additionally, we investigated the correlation between the PVS score and clinical features, offering utility in distinguishing SCA3 from healthy individuals, and the BG-PVS score, which provides a more comprehensive representation, with clinical indicators.

## Data Availability

The datasets generated and analyzed in this study are not publicly available to assure participant privacy. The datasets can only be provided upon reasonable request to the corresponding author.

## Abbreviations

Abbreviation: Full name
SCA3: Spinocerebellar ataxia type 3
PVS: The perivascular space
CSO: The center of the semiovoid
BG: Basal ganglia
MRI: Routine magnetic resonance imaging
AD: Alzheimer’s disease
HD: Huntington’s disease
PD: Parkinson’s disease
MS: Multiple sclerosis
CNS: Central nervous system
SARA: the Scale for the Assessment and Rating of Ataxia
ICARS: the International Cooperative Ataxia Rating Scale
PCR: Polymerase Chain Reaction
ROC: Receiver Operating Characteristic
AUC: the Area Under the Curve
BBB: Blood-brain barrier
CVR: cerebrovascular reactivity
ISF: Interstitial fluid
